# Hitchhiking with Nature: Snake Venom Peptides to Fight Cancer and Superbugs

**DOI:** 10.3390/toxins12040255

**Published:** 2020-04-15

**Authors:** Clara Pérez-Peinado, Sira Defaus, David Andreu

**Affiliations:** Department of Experimental and Health Science, Universitat Pompeu Fabra, Barcelona Biomedical Research Park, 08003 Barcelona, Spain; clara.perez@upf.edu (C.P.-P.); sira.defaus@upf.edu (S.D.)

**Keywords:** snake venoms, antimicrobial peptides, anticancer peptides, cathelicidin, defensin, crotamine, snake venom proteins, snake venom peptides

## Abstract

**Abstract:**

For decades, natural products in general and snake venoms (SV) in particular have been a rich source of bioactive compounds for drug discovery, and they remain a promising substrate for therapeutic development. Currently, a handful of SV-based drugs for diagnosis and treatment of various cardiovascular disorders and blood abnormalities are on the market. Likewise, far more SV compounds and their mimetics are under investigation today for diverse therapeutic applications, including antibiotic-resistant bacteria and cancer. In this review, we analyze the state of the art regarding SV-derived compounds with therapeutic potential, focusing on the development of antimicrobial and anticancer drugs. Specifically, information about SV peptides experimentally validated or predicted to act as antimicrobial and anticancer peptides (AMPs and ACPs, respectively) has been collected and analyzed. Their principal activities both in vitro and in vivo, structures, mechanisms of action, and attempts at sequence optimization are discussed in order to highlight their potential as drug leads.

**Key Contribution:**

This review describes the state of the art in snake venom-derived peptides and their therapeutic applications. This work reinforces the potential of snake venom components as therapeutic agents, particularly in the quest for new antimicrobial and anticancer drugs.

## 1. Introduction

Snakes are arguably among if not the most despised creatures in the entire animal kingdom. With some exceptions (serpents protected and revered, even worshipped, in ancient and some contemporary societies), for the vast majority of people, snakes epitomize harm, evil, and treachery. Examples are found in scriptures and ancient tradition (the serpent of Eden, the popular belief of Cleopatra’s asp-assisted suicide), classic literature (e.g., Poe’s *The cask of amontillado*, Doyle’s *The Speckled Band*) or colloquial language (“he’s a snake in the grass”). Beside this general perception of snakes incarnating terror and deceit, the ancient view of the serpent as a dual archetype of good and evil (e.g., Moses’ brass serpent, the rod of Asclepius, Hindu mythology) has not only survived, but paradoxically, become increasingly supported by evidence that, when properly used, snake venom (SV) compounds can actually help save lives rather than ending them [1].

SVs are cocktails of toxins refined over millions of years of evolution, sometimes encoding several powerful biological effects in a single component. SVs should, therefore, be considered as rich libraries of bioactive molecules with potential to treat human disorders. A pioneering example was the development of Captopril^®^ from a peptide discovered in the venom of the Brazilian pit viper *Bothrops jararaca* [2,3], and subsequent development of angiotensin-converting enzyme (ACE) inhibitors that have had a significant impact in hypertension control since the 1980s. Similarly, leads for other SV-derived drugs are mostly peptides and enzymes (Table 1). To date, most SV-based medicines have been approved for cardiovascular maladies or as diagnostic tools for blood-related abnormalities (Table 1). On the other hand, new uses currently being investigated in clinical and preclinical trials focus on other conditions, such as sclerosis, chronic pain, infections, or cancer (Table 1), thus revealing the potential of snake toxins to serve as molecular scaffolds in diverse therapeutic fields.

Here we focus on the potential relevance of SV compounds to two major therapeutic challenges of the 21st century: multi-drug-resistant bacteria and cancer. First, we are frightfully close to running out of effective antibiotics in the war against pathogenic bacteria, due to factors such as over-prescription, non-compliance, veterinary use, etc., fueling the emergence of superbugs [4,5] Recent forecasts of human costs associated with antimicrobial resistance are sobering: by 2050, the number of deaths attributed to antimicrobial resistance will increase from the current 700,000 a year to 10 million, becoming the main cause of death [6,7]. Second, new cancer therapies are urgently needed to complement and/or replace current chemotherapeutic drugs with their numerous contraindications. Cancer is now the second leading cause of death worldwide, accounting for one out of six deaths [8]. Annual statistics from the Global Cancer Observatory reported 18 million new cases and 9.6 million deaths in 2018, with the annual incidence expected to rise to 29.5 million cases and 16.4 million deaths globally by 2040 [9,10,11]. In light of the foregoing, the present work reviews SV-derived antimicrobial and anticancer peptides (SV-AMPs, and SV-ACPs, respectively), examining their biological activities, selectivity, structures, mechanisms of action, and overall therapeutic potential.

## 2. Antibacterial and Antitumoral Activity of Snake Venoms (SVs)

Envenomation is associated with a remarkably low incidence of microbial infections [18]. This is paradoxical if one considers that snakebites are puncture wounds. Thus, it is reasonable to suspect that SVs contain antibacterial agents [19]. Indeed, whole crotalid venoms possess antimicrobial activity against bacteria commonly found in snake oral cavities, such as *Pseudomonas aeruginosa* (minimal inhibitory concentration (MIC) = 80–160 μg/mL) and *Alcaligenes faecalis* (MIC = 5–20 μg/mL), and also toward human pathogens such as *Staphylococcus aureus* (MIC = 5–40 μg/mL) and *Escherichia coli* (MIC = 80–160 μg/mL) [20]. Additional studies demonstrated similar effects of SVs from a large variety of snakes, including viperids (*Agkistrodon rhodostoma*, *Bothrops atrox*, *B. jararaca, Bothrops alternatus* and *Daboia russellii russellii,* MICs <20 μg/mL) and elapids (*Pseudechis australis,* MIC = 40 μg/mL) [2,21,22,23].

In the late 1980s, elapid, crotalid, and viperid venoms were found to act against melanoma and chondrosarcoma cell lines [24]. Venoms of *B. jararaca* and *Crotalus durissus terrificus* also exhibited antitumoral properties, presumably by direct action on tumor cells and by modulating inflammatory responses [25,26]. *Ophiophagus hannah* venom demonstrated strong anti-cancer properties in vitro against pancreatic tumor cells, as well as anti-angiogenic activity in vivo [27].

Due to the therapeutic potential of SVs, efforts were focused on isolating and characterizing active compounds responsible for antibacterial and antitumoral activities. Examples included enzymes and proteins, the principal functions of which are not directly related to microbial or tumor clearance, but which nevertheless demonstrate antimicrobial, antifungal, antitumoral, and immunomodulatory properties. This is the case of L-amino acid oxidase (LAAO) and phospholipases A_2_s (PLA_2_s) [28,29]. Other SV components displaying various activities include metalloproteinases [30,31,32], cardio-, neuro- or myotoxins [33,34,35,36], disintegrins [37] and lectins [38,39,40], among others [41,42,43]. These compounds exhibit various mechanisms of action, including direct toxic action (PLA*_2_*s), free radical generation (LAAO), induction of apoptosis (PLA*_2_*s, LAAO and metalloproteinases), and anti-angiogenesis (disintegrins and lectins) [41]. SV-derived cationic AMPs and ACPs complete this arsenal of anti-infective and antitumoral components [44,45].

### 2.1. Snake Venom-Derived Antimicrobial and Anticancer Peptides (SV-AMPs and ACPs)

**Figure 1:**
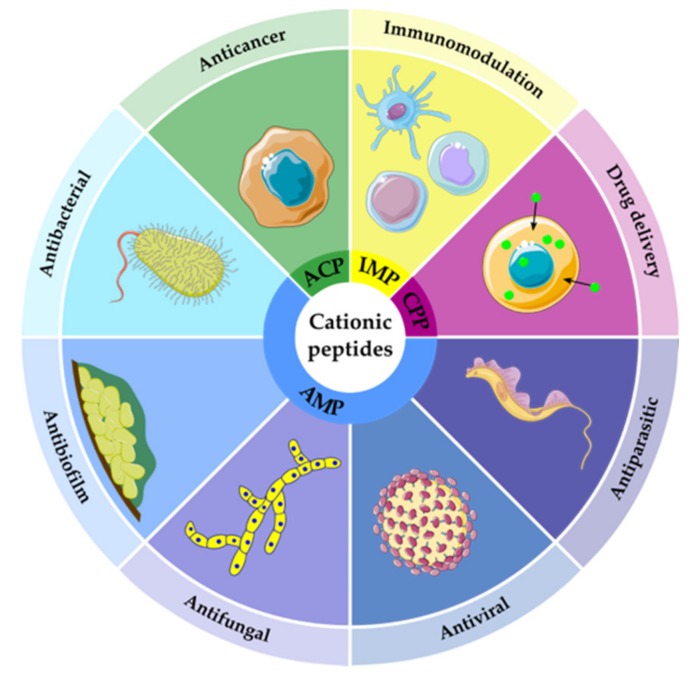
Principal functions of cationic peptides. ACP, anticancer peptides; AMP, antimicrobial peptides; CPP, cell-penetrating peptides; IMP, immunomodulatory peptides. This figure was prepared using the image repository Smart Servier Medical Art (available at: https://smart.servier.com).

Cationic peptides, including AMPs and ACPs, encompass a sequence-diverse family of peptides characterized by a net positive charge and a high content of hydrophobic residues [46]. The initial function proposed for cationic peptides was to act as AMPs against a broad spectrum of Gram-positive and Gram-negative bacteria, fungi, and parasites [47,48]. Subsequent studies have demonstrated antiviral and anti-biofilm properties and anticancer and immune modulatory activities [49,50]. Due to their ability to translocate across lipid membranes, cationic peptides have also been exploited as delivery vectors (Figure 1).

In particular, AMPs and ACPs contribute to microbe/tumor cell clearance by three complementary means: (i) direct membrane disruption; (ii) interference with key intracellular processes such as nucleic acid and protein synthesis; (iii) immune-cell function recruitment or activation via a broad array of functions, with the ultimate goal of clearing pathogens or tumor cells [51,52,53,54,55,56]. In addition, anti-angiogenesis [57,58] and suppression of metastasis [59] also contribute to tumor control by ACPs. Naturally occurring cationic peptides are present in every kingdom and phylum, including plants [60], animals [61], fungi [62] and bacteria [63], and they have also been identified in SV.

#### 2.1.1. SV-Cathelicidins (SV-CATHs)

**Figure 2:**
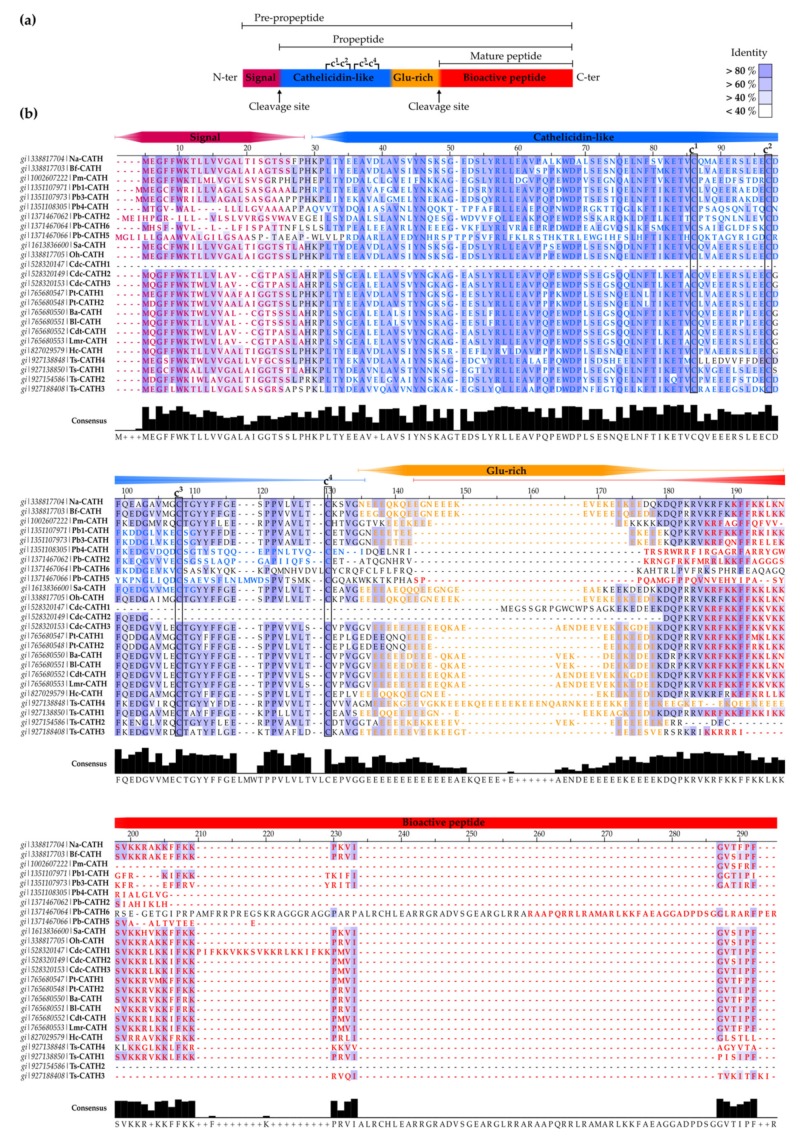
Snake-derived CATHs. (**a**) Schematic representation of CATH precursor structure. Domains are depicted in different colors and cleavage sites are highlighted. The Cys-pairing pattern is also annotated. (**b**). Alignment of CATH precursor sequences. Extended sequence information is available in Table S1. This multiple sequence alignment was performed using Clustal Omega [115] and represented with Jalview v2.11.0 software [116]. Residues were colored to match domains displayed in (**a**). Domain annotation was done using UniProt [117], National Center for Biotechnology Information (NCBI, https://www.ncbi.nlm.nih.gov/protein) or by homology (Table S1). The background is colored according to the percentage of residues in agreement with the consensus sequence appearing at the bottom.

Possibly the largest family of SV-AMPs and -ACPs described to date are the cathelicidins (CATHs), a group of structurally diverse bioactive peptides with antimicrobial, anticancer, and immunomodulatory functions, acting as effector molecules of the innate immune system [64,65]. Members of the CATH family possess highly homologous pre- and pro-regions comprising the N-terminal signal peptide and the cathelin (cathepsin L inhibitor)-like domain [66,67] (Figure 2a). In contrast, the C-terminal domain, which encodes the mature bioactive peptide, is diverse in amino acid (aa) sequence and higher structure [66,67].

While most mature CATHs are linear, 25–35-residue, amphipathic α-helical peptides, some family members (protegrins) are smaller, 12–18-residue peptides, displaying β-hairpin structures stabilized by disulfide bonds. Others consist of sequences enriched in specific aa, such as Trp-rich indolicidin [68]. Despite this conformational and compositional variability, most CATHs share certain physicochemical properties, including a generally positive charge and amphipathicity.

CATHs have been found in humans (LL-37) and other mammals [69,70,71,72,73,74], and in fish, birds, or reptiles [75,76,77]. SV-derived CATHs (SV-CATHs) were first identified by Zhao et al. in 2008 from elapid venom gland cDNA libraries [76]. To date, 25 SV-CATHs have been identified, as well as fragments derived from parental SV-CATHs (see Table 2 for a summary of mature SV-CATHs and their principal activities, and Table S1 for SV-CATH precursors).

Overall, SV-CATH precursors range from 184 to 194 aa, with some exceptions (Table S1). The signal peptide domain comprises the ~22 N-terminal aa residues, followed by 65–66 aa residues forming the cathelin domain. The 30–34 C-terminal aa residues encode the mature peptide, sometimes preceded by a Glu-rich domain of 9 to 29 aa, responsible for inactivation of the mature peptide, by inducing conformational changes [78]. CATHs from *Python bivittatus*, *Thamnophis sirtalis*, and *Protobothrops mucrosquamatus* exhibit exceptions to the aforementioned domain features, presenting different lengths or even being absent in some cases (Table S1).

Snake-derived CATH precursors generally display >50% sequence identity, excluding four of the six CATHs predicted from the boid, *P. bivittatus* (Pb-CATH 2, Pb-CATH 4, Pb-CATH 5 and Pb-CATH 6), and one predicted from the colubrid, *T. sirtalis* (Ts-CATH 4) (Figure S1). Sequence analysis (Figure 2b) reveals conserved residues in the N-terminal part of the signal domain, as well as most of the cathelin domain and in the C-terminal end encoding the mature active peptide. Table 2 summarizes the principal biological activities of mature SV-CATHs and Table 3 compares experimentally validated SV-CATHs in terms of biological activity, hemolysis, and selectivity for microbial/tumor cells, rather than healthy eukaryotic cells.

- **Oh-CATH:** identified in the venom gland of the king cobra (*O. hannah*), this was the first predicted SV-CATH to be synthesized and experimentally validated as an AMP [76] (Table 2). Oh-CATH exerts strong salt-resistant, antibacterial activity against Gram-positive and Gram-negative bacteria (MICs in the 1–20 µg/mL range) with weak hemolysis (~10% hemolysis observed at 200 µg/mL) [76,79]. It is apparently membrane-active and is an inhibitor of ATP-synthase [80,81]. A collection of analogs designed by Zhang et al., to study the structure-function relationships of Oh-CATH, suggested that the four N-terminal aa residues are responsible for cytotoxicity toward eukaryotic cells, while the C-terminal 10 strongly influence antimicrobial activity [79]. Accordingly, OH-CATH30, the most promising analog, lacking the four N-terminal aa, also named OH-CATH(5-34), was further characterized and optimized. Then, OH-CATH30 was tested against a panel of 584 clinical isolates of 14 different species, showing antibacterial activity against 85% of them and overall higher efficacy against Gram-positive strains [82].

Li et al. successfully downsized OH-CATH30 by removing the 10 C-terminal aa and by including single-aa mutations, giving rise to OH-CM6, which displays almost identical activity against a panel of Gram-positive and Gram-negative bacteria [81]. Although OH-CATH30 and OH-CM6 displayed potent antibacterial activity (MICs from 1.56 to 12.5 μg/mL against clinical isolates from *E. coli*, *P. aeruginosa* and methicillin-resistant *S. aureus* (MRSA)), both were inactive against *Candida albicans* strains (MICs >200 μg/mL) [81]. L-forms of both peptides conserve some antimicrobial activity in the presence of 25% serum, probably due to the membrane-targeting mechanism before enzymatic degradation occurs, but pre-incubation in 100% serum results in total activity loss after 4 h. In contrast, activity of their D-amino acid forms remained unchanged even after 12 h pre-incubation in 100% serum [81]. In vivo studies carried out by the same group revealed that intraperitoneal (*i.p.*) LD_50_s of OH-CATH30 and OH-CM6 in mice were 120 and 100 µg/g, respectively. Both peptides were able to rescue infected mice in a bacteremia model induced by drug-resistant *E. coli* at 10 µg/g as well as decreased TNF-α production in a mouse model of neutropenic thigh infection [81].

- **Na-CATH:** this CATH from the venom gland of the Chinese cobra *N. atra* [76] also demonstrated powerful, salt-resistant, antimicrobial activity against Gram-positive and Gram-negative bacteria, including *Francisella novicida* (the non-virulent strain in humans related to *Francisella tularensis*, the causative agent of tularemia) [98], *E.coli, Aggregatibacter actinomycetemcomitans*, *Bacillus cereus* [99], *P. aeruginosa* [100], and *S. aureus* [101] at low concentrations (EC_50_ < 3 µg/mL). Na-CATH is also active against *Mycobacterium smegmatis* [102], *Burkholderia thailandensis* (closely related to *B. pseudomallei*, the causative agent of melioidosis) [103] and *Bacillus anthracis* (anthrax) [104]. The last two strains are of particular relevance due to their potential use as biological weapons. Indeed, in vivo studies using wax moth larvae demonstrated that Na-CATH was able to rescue 100% of waxworms after *B. anthracis* Sterne infection at low peptide concentrations [104]. In addition, Na-CATH is not only active against planktonic bacteria, but also inhibits *S. aureus* and *B. thailandensis* biofilm formation [101,102], while inducing minimal hemolysis (<2% at 100 µg/mL) [99]. However, Na-CATH did not inhibit *Pseudomonas* biofilm formation [100].

Structurally, Na-CATH folds into a well-defined amphipathic α-helix between residues Phe3 and Lys23 in the presence of trifluoroethanol. The remaining 11-residue tail, consisting mostly of aromatic and hydrophobic residues, does not present a defined structure, but appears to interact with lipid membranes [118]. Na-CATH contains an 11-residue sequence [KR(F/A)KKFFKK(L/P)K] known as an ATRA motif, repeated twice and almost totally shared by other SV-CATHs (Table 2). The ATRA motif, when tested by itself, is also active, but the Pro at position 10 dramatically reduces its antibacterial potency, probably by destabilizing its helical structure, while a Phe to Ala change at position 3 does not impair activity [98,99].

Du et al. postulated that Na-CATH is able to disrupt bacterial membrane-like liposomes via membrane thinning or transient-pore formation [118]. This hypothesis was further confirmed in vitro by Gupta el al. and Juba et al., who described membrane depolarization and transient-pore formation in *Mycobacterium smegmatis* [103], as well as in *E.coli* and *B. cereus* [119] after Na-CATH treatment. However, Samuel et al. suggested a more detailed mechanism changing from membrane disruption to pore-based lysis, depending on liposome lipid composition and phase [120].

- **Bf-CATH:** unlike other 34-aa AMPs predicted as SV-CATHs, the purified peptide from *Bungarus fasciatus* venom gland is a 30-aa peptide lacking the four N-terminal residues (Table 2). This difference in length suggests different enzymatic processing rather than the proposed elastase-like protease cleavage at the conserved site (Val) or post-processing of the 34-residue precursor [97]. Expression of Bf-CATH is widespread, including stomach, trachea, skin, muscle, heart, kidney, lung, brain, intestine, spleen, liver, ovary and venom glands [97].

Like other SV-CATHs, Bf-CATH has a random-coil conformation in aqueous solution, but adopts an α-helical structure in hydrophobic or membrane-like environments, specifically from residues Phe2 to Phe18 [97]. Bf-CATH presents potent antimicrobial activity against a broad range of clinically isolated, drug-resistant Gram-negative and Gram-positive bacteria, as well as saprophytic fungi [97]. In vitro and in vivo analyses demonstrate that Bf-CATH partially retains antibacterial activity in the presence of human serum, but not in gastrointestinal fluids. Its fluorescein-labeled analog can be absorbed into the mouse circulatory system within 30 min after intraperitoneal injection, without tissue accumulation (totally excreted after 24 h) [121]. Interestingly, Bf-CATH was less prone to induce bacterial resistance than classical antibiotics, such as ciprofloxacin or gentamicin, when administered at sublethal concentrations [94]. Bf-CATH demonstrated membrane disruptive antibacterial activity [94], as well as the capacity to inhibit secretion of pro-inflammatory molecules, such as TNF-α, IL-8, IL-1, or MCP-1 (monocyte chemoattractant protein-1) and to inhibit O_2_·^–^ production induced by *Propionibacterium acnes* (acne vulgaris) [95].

In vivo studies by Wang et al. showed an overall anti-inflammatory effect of Bf-CATH and reduced *P. acnes*-induced granulomatous inflammation, revealing its therapeutic potential to treat acne [95]. In vivo*,* Bf-CATH reduced bacterial loads and colonization in murine models of *P. aeruginosa* and *Salmonella typhimurium* infections, attenuating symptoms and intestinal alterations [94,121]. Bf-CATH also demonstrated in vivo prevention of intestinal barrier dysfunction in mouse and piglet models of lipopolysaccharide-induced endotoxemia/inflammation, presumably by downregulating TNF-α expression through the NF- κB signaling pathway [89,92]. Parallel downregulation of NF- κB signaling and activation of the signal transducer and activator of transcription 1 (STAT-1) in weanling piglets seems to help suppress intestinal inflammation and to enhance phagocytosis of immune cells, and modulation of intestinal immune responses during stress-related inflammatory processes, such as weaning [91]. A recent study by Liu et al. suggested that pre-treatment with Bf-CATH ameliorates *P. aeruginosa*-induced pneumonia by enhancing NETosis (activation and release of neutrophil extracellular traps), confirming the immunomodulatory activity of Bf-CATH [88].

Anti-tumor activity of Bf-CATH was also investigated, showing potent in vitro activity against mouse melanoma cells (IC_50_ ~7 μM) and in vivo inhibition of mouse melanoma cell proliferation, migration, and angiogenesis, but with a negligible effect against human tumoral cell lines (IC_50_s from ~20–100 μM). Its anti-tumor mechanism is related to membrane permeabilization, and to DNA binding and prevention of vascular endothelial growth factor (VEGF) gene expression [93].

Different strategies have addressed structure-activity relationships of Bf-CATH to optimize its activity [86,87,96,97,124,125,126,127]. For instance, the 15-aa BF-15, mostly retains the antimicrobial activity of Bf-CATH, permeabilizes membranes, and is more stable in serum than the native peptide [96,97]. Cbf-K16, a Bf-CATH analog obtained from substitution of Glu16 ➝ Lys, also showed antibacterial activity against a recombinant New Delhi metallo-beta-lactamase-1 (NMD-1)-carrying *E. coli* strain, as well as improved anti-tumor activity against human and mouse lung carcinoma cells, compared to Bf-CATH, both effects presumably achieved by membrane disruption and DNA binding [125,126]. Trp/Arg-rich analogs of Bf-CATH were also designed, from which ZY13, the most potent and least hemolytic analog, exhibited in vitro antibacterial and promising antifungal and anti-inflammatory properties both in vitro and in a mouse *C. albicans*-induced vaginitis model [87].

Finally, Bf-CATH production and delivery systems were investigated. Bf-CATH encapsulation was tested in poly(D,L-lactide-co-glycolide) (PLGA) microspheres and poly(ethylene glycol)-poly(lactic-acid-co-glycolic acid) block copolymers (4-arm-PEG-PLGA). Both systems retained the antibacterial activity of the free antimicrobial peptide and released Bf-CATH over >15 days [128,129]. Different recombinant DNA strategies were also reported to effectively produce Bf-CATH, such as small ubiquitin-related modifier (SUMO) technology or intein-based technology, both expressed in *Bacillus subtilis* (achieved peptide yields of ~3 mg/L and 0.5 mg/L, respectively) [130,131].

- **Cdt-CATH:** also named as crotalicidin (Ctn), is a 34-aa peptide from the South American rattlesnake (*Crotalus durissus terrificus*) venom. Among SV-CATHs identified in pit vipers by Falcão et al. [84] (lutzicidin, lachesicidin, batroxicidin, collectively named vipericidins), Ctn has been the most studied, showing potent bactericidal effects against Gram-negative and Gram-positive bacteria (MICs <10 μM) [84], anti-parasitic (anti-trypanosomatid) activity [109], and activity against opportunistic yeasts and dermatophytes, alone or in combination with conventional antifungals [108]. In addition, Ctn has shown potent anti-tumor activity against different leukemia cell lines (IC_50_s <5 μM) [107].

The general mechanism of Ctn against bacteria or parasites is membrane-related and its ability to interfere and disrupt biological membranes have been extensively described [109,122]. Ctn toxicity to leukemia cells is also linked to its membranolytic effect, although it also seems able to interfere with key intracellular pathways, ultimately contributing to tumor cell death (Pérez-Peinado et al., submitted). In addition to the direct cytotoxic effect, the immunomodulatory properties of Ctn have been explored, showing an overall pro-inflammatory profile in the presence of heat-inactivated bacterial antigens and IFN-γ [110], contrasting with general anti-inflammatory behavior of other SV-CATHs.

**Figure 3:**
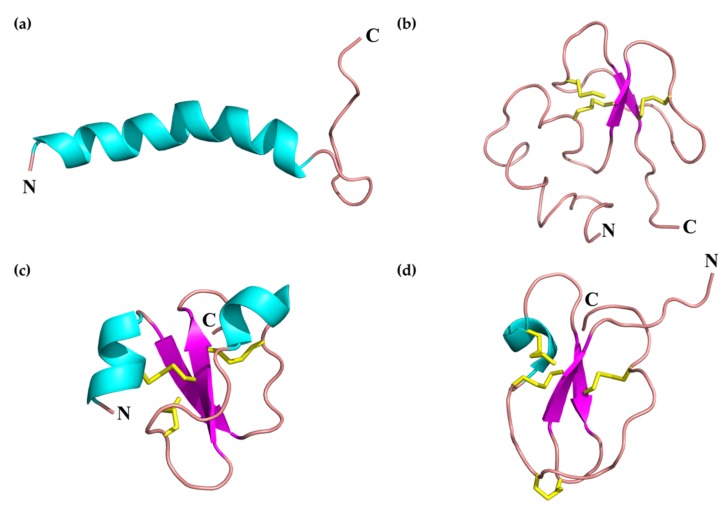
Three-dimensional structures adopted by SV-AMPs and -ACPs. (**a**) crotalicidin (Ctn), PDB 2MWT. (**b**) Cdt-defensin, 3D structure prediction obtained using the Iterative Threading ASSEmbly Refinement (I-TASSER) web server [136] (available at: https://zhanglab.ccmb.med.umich.edu/I-TASSER/). (**c**) Crotamine, PDB 4GV5. (**d**) Omwaprin, PDB 3NGG. Representation was performed using the PyMOL Molecular Graphical System, Version 2.0. Schrödinger, LLC [137]. Color code: blue for α-helix, magenta for β-sheet and pink for, loops. Disulfide pairing is also indicated in yellow.

From a structural viewpoint, circular dichroism and nuclear magnetic resonance (NMR) studies indicate that Ctn is fully in a random-coil conformation in aqueous solution, but may change its structure in membrane-like environments (i.e., dodecylphosphocholine micelles), displaying an α-helix conformation at the N-terminal end (residues 3 – 21) plus a C-terminal random coil tail (Figure 3a) [107]. The Ctn framework is quite similar to those proposed for Na-CATH and Bf-CATH (N-terminal α-helix plus a random coil C-term end), suggesting a common template shared by SV-CATHs.

A rational dissection of Ctn involving in silico enzymatic cleavage was performed by Falcao et al. in order to define a shorter, active motif [107]. Two fragments resulted from cleavage at Val14: Ctn [1–14] and Ctn [15–34]. Surprisingly, the former was inactive, regardless of its amphipathic α-helical conformation. In contrast, the latter preserved some of the activity of Ctn, despite its overall disordered structure. In fact, Ctn [15–34] presents lower hemolytic and cytotoxicity effects than its predecessor Ctn, despite improved selectivity for Gram-negative bacteria and enhanced stability in human serum, presumably due to serum protein binding and its preferred scaffold [107,132,133]. Ctn [15–34] lost activity against dermatophytes, but had enhanced activity against pathogenic yeasts, such as several (multi-resistant) *Candida* species, and acted synergistically with amphotericin B [108,134]. Although Ctn was also able to induce necrosis of all developmental forms of *T. cruzi* (the Chagas’ disease agent), Ctn [15–34] only retained activity against the trypomastigote form [109]. The fragment also showed antiviral activity against the infectious myonecrosis virus, an epizootic agent that threatens shrimp production in Brazil, and for which no current treatment exists [135]. Like Ctn, Ctn [15–34] acts via membrane permeabilization and necrosis, as described for strains of *E.coli* and *C. albicans* [122,123].

Insights into the functionality of structural domains of SV-CATH were presented by Oliveira-Júnior et al. using Ctn [15–34] as a model. As described above, SV-CATHs contain an anionic region between the cathelin domain and the mature AMP domain, not usually found in other CATHs. Thus, by including a Glu decapeptide at the N-terminus of Ctn [15–34] to generate a “pro-peptide” model, Oliveira-Júnior et al. confirmed that this acidic moiety contributes to a more helical conformation and prevents peptide antimicrobial activity before its release [78].

- **Other snake-derived CATHs**: additional pit viper-derived CATHs were predicted by Falcao et al., from the venom gland of *Lachesis muta rhombeata* (lachesicidin), *Bothrops atrox* (batroxicidin) and *B. lutzi* (lutzicidin), as well as two clones from the elapid, *Pseudonaja textili*s (Pt-CATH1 and Pt-CATH2). Batroxicidin and Pt-CATH1 displayed antibacterial potency comparable to that of Ctn, but were more hemolytic [84]. Moreover, batroxicidin induced *T. cruzi* cell death by membrane disruption and showed an overall proinflammatory profile [110,111].

CATHs were similarly predicted/identified in the genome of the boid, *P. bivittatus*, both by Kim et al. and Cai et al. (Table 2), denominated Pb-CATH1-5 and CATHPb1-6, respectively [112,113]. From the set of Pb-CATHs identified by Kim et al., three (Pb-CATH1, Pb-CATH3 and Pb-CATH4) encode mature AMPs displaying powerful antibacterial activity against Gram-negative bacteria (MICs from 0.5 to 8 μg/mL) [113]. Pb-CATH4, for instance, induced bacterial death by toroidal pore formation and displayed low hemolysis and cytotoxicity, as well as considerable stability in serum [113]. In parallel, CATHPb1 exhibited protection in mice infected with MRSA and VRSA (vancomycin-resistant *S. aureus*), via neutrophil-mediated bacterial clearance and immunomodulation employing Mitogen-Activated Protein Kinases (MAPKs) and NF- κB pathways [112].

Finally, another CATH-related peptide, Hc-CATH (Table 2), was identified in the genome of the sea snake *Hydrophis cyanocinctus* (annulated sea snake) [105]. Unlike the overall preference of terrestrial snake-derived CATHs for Gram-negative bacteria, Hc-CATH displays more or less equal activity against both Gram-negative and Gram-positive bacteria as a result of membrane permeabilization [105]. Hc-CATH has intrinsic structural advantages compared to other CATHs, such as high stability in the presence of salts, high temperature (≤90 °C), and serum proteases, together with low toxicity against eukaryotic cells [105]. In a recent study, Carlile et al. demonstrated anti-inflammatory properties and bacterial load reduction by Hc-CATH in vivo, using wax moth and mouse models of intraperitoneal and respiratory infection induced by *P. aeruginosa* [106].

#### 2.1.2. SV-Defensins

**Figure 4:**
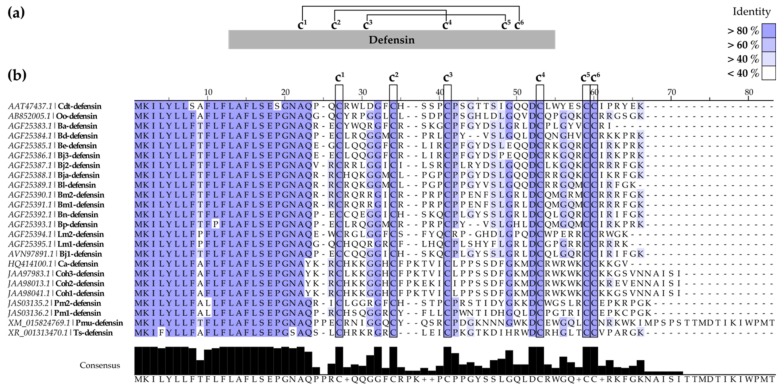
SV-defensins. (**a**) Schematic representation of general SV-defensin structure highlighting the Cys-pairing pattern. (**b**) Alignment of SV-defensin sequences. Multiple sequence alignment was performed using Clustal Omega [115] and represented with Jalview v2.11.0 software [116]. Background is colored according to the percentage of identity (residues in agreement with the consensus sequence depicted at the bottom).

Together with CATHs, defensins are a major group of host defense peptides found in vertebrates and invertebrates, exhibiting broad-spectrum activity against bacteria, fungi, and enveloped viruses [138]. Defensins are cationic, Cys-rich peptides of 3.5–6 kDa, with a typical β-sheet-rich folding stabilized by three disulfide bonds (Figure 3b). Defensins are divided into three major groups based on length and Cys-pairing: α-, β-, and θ-defensins [139]. Although defensins have been deeply studied in mammals, little information is available for those of SV origin. More than 20 β-defensin-like sequences have been described in snakes [140,141,142], half of them identified in the genus *Bothrops* (Table S2). However, no details regarding their antibacterial or antitumoral potency are available. Based on the sequence alignment presented in Figure 4, SV defensins present a highly conserved N-terminal region, as well as conserved Cys residues in disulfide bonds. Gly, Pro, and Asp residues are also highly conserved.

Structurally, crotamine is closely related to β-defensins, displaying an α_1_β_1_α_2_β_2_ arrangement, with the whole structure stabilized by three disulfide bonds [143] (Figure 3c). Crotamine is a 42-aa neuro- and myotoxic peptide, initially isolated from the South American rattlesnake (*C. durissus terrificus*). Crotamine’s anti-infective, antitumoral, and cell-penetrating properties have been deeply characterized both in vitro and in vivo [144]. Crotamine displays modest antibacterial activity (MICs in the 25–100 μg/mL range against *E. coli* strains) and does not induce hemolysis at high concentrations (no hemolysis observed up to 1024 μg/mL) [145]. Additional studies revealed antifungal activity of crotamine (12.5–50 μg/mL) against *Candida* spp., including clinically resistant strains [146]. The anticancer potential of crotamine has also been studied in vitro and in vivo, showing selective cytotoxicity against tumor cell lines at low concentrations (~5 μg/mL) and significant inhibition of tumor growth and increased lifespan in a melanoma mouse model [147,148].

Derivatives of crotamine [CyLoP-1 (cytosol localizing peptide 1) and the NrTPs (nucleolar-targeting peptides)] have been designed, with cytosolic and nucleolar localization, respectively, instead of the nuclear distribution pattern of crotamine [149,150,151].

#### 2.1.3. Waprins

Waprins are a family of ~50-residue, Cys-rich peptides first isolated from the venom of *Naja nigricollis* (black-necked spitting cobra), named nawaprin (Naw), and subsequently in venom of the Indian taipan (*Oxyuranus microlepidotus*), named omwaprin (Omw) [152,153]. Omwaprin displays salt-resistant antibacterial activity against Gram-positive bacteria such as *Bacillus megaterium* and *Staphylococcus warneri* [153]. In vivo experiments in mice demonstrated that Omw is non-toxic at doses up to 10 µg/g after *i.p.* injection [153]. Tertiary structure of Omw and Naw has been studied by X-ray and NMR, respectively, showing a complex disc-like shape with four disulfide bonds [152,154] (Figure 3d).

Two derivatives, Omw1 and Omw2, also present modest antimicrobial, antifungal, and antibiofilm activity at concentrations from 16–500 μg/mL, presumably due to membrane disruption with low hemolysis induction in the same concentration range (~10 % hemolysis at 250–500 μg/mL) [155].

## 3. Conclusions

Despite major advances, important challenges remain in the anti-infective and anticancer drug discovery pipeline. The emergence of superbugs has created an antibiotic resistance crisis, evidencing the need for new anti-infective strategies. Paradoxically, investment and R&D by the pharmaceutical industry in this area is losing momentum [156,157]. In parallel, although meaningful progress has been made in cancer research, new therapeutic alternatives are still required in order to avoid undesired effects linked to current therapies and to target multidrug-resistant cells [158,159].

In this context, peptide-based therapeutics emerge as a feasible alternative to traditional anti-infective and cytotoxic drugs. Examples of promising natural AMPs and ACPs (e.g., cecropins, magainins, defensins) have been described since the 1980s. Study of SV-derived AMPs and ACPs, has been limited to the last decade, but thorough work has been done recently to search for ideal candidates. We expect to see SV-derived AMPs and ACPs in the pharmaceutical market in the near future. Although peptides still face important challenges (such as potential toxicity/immunogenicity, non-oral activity, limited bioavailability, cost, etc.) that hinder their penetration of the market, significant advances have been made to overcome these hurdles (e.g., rational design, cyclization, peptidomimetics, conjugation to macromolecules/polymers/delivery vectors, etc.).

Snake-derived AMPs and ACPs presented here are but a small number of the plethora of experimentally validated AMPs (~2800 sequences [160]) and ACPs (~500 [161]) reported to date. Throughout this manuscript, we have described their in vitro and in vivo validation and bioavailability, and have mentioned strategies for their optimization (downsizing, conjugation to polymers, etc.) and even some scale-up attempts (for Bf-CATH, for instance). Taken together, these advances testify to the significant potential of SV peptides as future anti-infective and antitumoral therapeutics and highlight the utility of snake venoms as a font for drug discovery. With so few snake venoms investigated to date and so many more waiting to be explored, the number of drug leads derived from snake venoms can only increase in the future.

## Figures and Tables

**Table 1:** Snake venom-derived products (drugs and diagnostic tools) currently on the market or in clinical and pre-clinical trials. Information was extracted from literature and updated on the corresponding company website and/or the U.S. National Library of Medicine (https://clinicaltrials.gov/ct2/home). *Withdrawn.

**Commercial Drugs** [12,13,14,15,16,17]				
**Drug (Commercial Name)**		**Source Organism**	**Target**	**Therapeutic Use**
Captopril (Capoten^®^)		*Bothrops jararaca*	Angiotensin-converting enzyme (ACE)	Hypertension
Enalapril (Vasotec^®^)		*Bothrops jararaca*	ACE	Hypertension
Tirofiban (Aggrastat^®^)		*Echis carinatus*	Glycoprotein IIb/IIIa	Acute coronary syndromes
Eptifibatide (Integrilin^®^)		*Sistrurus miliarius barbouri*	Glycoprotein IIb/IIIa	Acute coronary syndromes
Batroxobin (Defibrase^®^)		*Bothrops* sp.	Fibrinogen	Infarction / Ischemia / Microcirculation dysfunctions
Platelet gel (Plateltex-Act^®^)		*Bothrops atrox*	Fibrinogen	Platelet-induced tissue-healing
Fibrin sealant (Vivostat^®^)		*Bothrops moojeni*	Fibrinogen	Autologous fibrin sealant in surgery
Haemocoagulase (Reptilase^®^)		*Bothrops atrox*	Fibrinogen Factor X / Prothrombin	Hemorrhage
Ximelagatran (Exanta^®^)*		Cobra venom	Thrombin	Atrial fibrillation / Blood clotting
Ancrod (Viprinex^®^)*		*Agkistrodon rhodostoma*	Fibrinogen	Heparin-induced thrombocytopenia
**Drugs in Clinical and Pre-Clinical Trials** [12,13,14,15,16,17]				
**Drug Name**	**Phase**	**Source Organism**	**Target**	**Therapeutic Use**
Fibrolase (Alfimeprase)*	III	*Agkistrodon contortrix*	Fibrinogen	Stroke and catheter occlusion
Crotoxin	I	*Crotalus durissus terrificus*	Unknown	Cancer
Cenderitide	II	*Dendroaspis angusticeps*	Natriuretic peptide receptor	Heart failure
RPI-MN	I	* Naja atra *	Nicotinic acetylcholine receptor	HIV / Amyotrophic lateral sclerosis / Herpes simplex keratitis
RPI-78M	I/II	* Naja atra *	Nicotinic acetylcholine receptor	Multiple sclerosis / Herpes simplex infections / Adrenomyeloneuropathy
RPI-78	preclinical	* Naja atra *	Nicotinic acetylcholine receptor	Pain / Rheumatoid arthritis
Prohanin	preclinical	*Ophiophagus hannah*	Nitric oxide synthase	Chronic pain
Oxynor	preclinical	*Oxyuranus scutellatus*	Unknown	Wound healing
Natriuretic peptides	preclinical	*Oxyuranus microlepidotus*	Natriuretic peptide receptor	Heart failure
Textilinin-1^TM^	preclinical	*Pseudonaja textilis*	Plasmin	Preoperative bleeding
Vicrostatin	preclinical	Chimeric; *Echis carinatus / Agkistrodon contortrix*	Integrin receptor	Cancer
Haempatch^TM^	preclinical	*Pseudonaja textilis*	Prothrombin	Blood loss during
				vascular trauma
CoVase^TM^	preclinical	*Pseudonaja textilis*	Factor Xa	Hemorrhage
Contortrostatin	preclinical	*Agkistrodon contortrix*	Integrin	Breast cancer
**Diagnostic Tools** [12,13,14,15,16,17]				
**Product**		**Source Organism**	**Target**	**Clinical Assessment of:**
Protac^®^		*Agkistrodon contortix*	Protein C activation	Protein C
Reptilase^®^		*Bothrops jararaca*	Fibrinogen	Fibrinogen
Ecarin clotting time		*Echis carinatus*	Prothrombin	Meizothrombin
Textarin^®^/Ecarin ratio		*Pseudonaja textilis*	Prothrombin	Lupus anticoagulant
Russell’s viper venom-factor X		*Daboia russelii*	Factor X	Factor X
Dilute Russell’s Viper Venom Time		*Daboia russelii*	Factor X, Factor V	Lupus anticoagulant
Taipan Venom Time		*Oxyuranus scutellatus*	Prothrombin	Lupus anticoagulant
Pefakit^®^ APCR Factor V Leiden		*Daboia russelii / Notechis scutatus scutatus*	Factor V / Protein C / Prothrombin	Resistance to activated protein C
Botrocetin^®^		*Bothrops sp*	Factor VIIIa	von Willebrand Factor

**Table 2:** Mature snake cathelicidins (CATHs) identified to date and their properties. Information was extracted from literature or the National Center for Biotechnology Information (NCBI). A unified name was given to each CATH, according to the binomial initials of the snake. Biological and hemolytic activities of experimentally validated CATHs. Hemolytic activity denotes causing 10% hemolysis (low: HC_10_ >50 μg/mL; medium, 50 > HC_10_ > 10 μg/mL; high, HC_10_ <10 μg/mL) n.d.: non detectable activity.

Source Organism	Unified Name	Common name	Mature Peptide Sequence	Length	Hemolysis	Activity	Ref
*Ophiophagus hannah*	Oh-CATH	KF-34	KRFKKFFKKLKNSVKKRAKKFFKKPRVIGVSIPF	34	Medium	G+ and G- bacteria.	[76,82,83,84]
*Bungarus fasciatus*	Bf-CATH	Cath-BF	KRFKKFFRKLKKSVKKRAKEFFKKPRVIGVSIPF	34	High	G+ and G- bacteria.	[85,86]
	Bf-CATH30	BF-30, cathelicidin-BF, C-BF, cathelicidin-WA, CWA	KFFRKLKKSVKKRAKEFFKKPRVIGVSIPF	30	High	G+ and G- bacteria, fungi and tumor cells. Anti-inflamatory. Activation of innate immunity.	[87,88,89,90,91,92,93,94,95,96,97]
*Naja atra*	Na-CATH	-	KRFKKFFKKLKNSVKKRAKKFFKKPKVIGVTFPF	34	Low	G+ and G- bacteria.	[98,99,100,101,102,103,104]
*Hydrophis cyanocinctus*	Hc-CATH	-	KFFKRLLKSVRRAVKKFRKKPRLIGLSTLL	30	Low	G+ and G- bacteria and fungi. Anti-inflamatory. Inactive against tumor cells.	[105,106]
*Crotalus durissus terrificus*	Cdt-CATH	crotalicidin, Ctn	KRFKKFFKKVKKSVKKRLKKIFKKPMVIGVTIPF	34	High	G+ and G- bacteria, fungi, parasites and tumor cells. Overall proinflammatory.	[84,107,108,109,110]
*Bothrops atrox*	Ba-CATH	batroxicidin, BatxC	KRFKKFFKKLKNSVKKRVKKFFRKPRVIGVTFPF	34	High	G+ and G- bacteria and parasites. Overall proinflammatory.	[84,110,111]
*Pseudonaja textilis*	Pt-CATH1	Pt-CRAMP1	KRFKKFFMKLKKSVKKRVMKFFKKPMVIGVTFPF	34	High	G+ and G- bacteria.	[84]
	Pt-CATH2	Pt-CRAMP2	KRFKKFFRKLKKSVKKRVKKFFKKPRVIGVTIPF	34	n.d.	n.d.	[84]
*Lachesis muta rhombeata*	Lmr-CATH	lachesicidin	KRFKKFFKKVKKSVKKRLKKIFKKPMVIGVTFPF	34	n.d.	n.d.	[84]
*Bothrops lutzi*	Bl-CATH	lutzicidin	KRFKKFFKKLKNNVKKRVKKFFRKPRVIGVTIPF	34	n.d.	n.d.	[84]
*Python bivittatus*	Pb-CATH1	CATHPb1	KRFKKFFRKIKKGFRKIFKKTKIFIGGTIPI	31	Low	G+ and G- bacteria and fungi. Chemotactic. Anti-inflammatory.	[112]
	∆Pb-CATH1	∆Pb-CATH1	RVKRFKKFFRKIKKGFRKIFKKTKIFIG	28	Medium	G+ and G- bacteria.	[113]
	Pb-CATH2	CATHPb2	KRNGFRKFMRRLKKFFAGGGSSIAHIKLH	29	Low	G+ and G- bacteria and fungi. Chemotactic. Weakly anti-inflammatory.	[112]
	∆Pb-CATH2	Pb-CATH3	HRVKRNGFRKFMRRLKKFFAGG	22	Medium or low	G+ and G- bacteria.	[113]
	Pb-CATH3	CATHPb3	KRFQNFFRELEKKFREFFRVYRITIGATIRF	31	Low	Inactive against G+ and G- bacteria and fungi. Immunomodulatory inactive.	[112]
	Pb-CATH4	CATHPb4	TRSRWRRFIRGAGRFARRYGWRIALGLVG	29	Medium or high	G+ and G- bacteria and fungi. Weakly anti-inflammatory.	[112]
	∆Pb-CATH4	∆Pb-CATH4	TRSRWRRFIRGAGRFARRYGWRIA	24	Medium	G+ and G- bacteria and tumor cells.	[113]
	Pb-CATH5	CATHPb5	SPPQAMGFPPQVNVEHYIPASYSVAALTVTEEE	33	Low	Inactive against G+ and G- bacteria and fungi. Immunomodulatory inactive.	[112]
	Pb-CATH6	CATHPb6	RAAPQRRLRAMARLKKFAEAGGADPDSGGLRARFPER	37	Low	Inactive against G+ and G- bacteria and fungi. Weakly anti-inflammatory.	[112]
*Sinonatrix annularis*	Sa-CATH	-	KFFKKLKKSVKKHVKKFFKKPKVIGVSIPF	30	Low	G+ and G- bacteria and fungi. Anti-inflamatory.	[114]
*Crotalus durissus cascavella*	Cdc-CATH1	Cas-CATH isoform 1	KRFKKFFKKVKKSVKKRLKKIFKKPIFKKVKKSVKKRLKKIFKKPMVIGVTIPF	54	n.d.	n.d.	NCBI
	Cdc-CATH2	Cas-CATH isoform 2	KRFKKFFKKVKKSVKKRLKKIFKKPMVIGVSIPF	34	n.d.	n.d.	NCBI
	Cdc-CATH3	Cas-CATH isoform 3	KRFKKFFKKVKKSVKKRLKKIFKKPMVIGVTIPF	34	n.d.	n.d.	NCBI
*Thamnophis sirtalis*	Ts-CATH1	-	KRFKKFFKKIKKSVKKRVKKLFKKPRVIPISIPF	34	n.d.	n.d.	NCBI
	Ts-CATH3	-	KKRRRIRVQITVKITFKI	18	n.d.	n.d.	NCBI
	Ts-CATH4	-	KKGLKKLFKRKKVVAGYVTA	20	n.d.	n.d.	NCBI
*Protobothrops mucrosquamatus*	Pm-CATH	-	KRFAGFFQFVVGVSFRF	17	n.d.	n.d.	NCBI

**Table 3:** Properties of mature snake-derived CATHs. Hemolytic activity (HC_10_, μg/mL) and biological activity (minimal inhibitory concentration (MIC) or IC_50_, μg/mL) against representative or clinically isolated (CI) microorganisms and tumor cells were collected from published sources. If more than one value was available in the literature, a range is given. Selectivity ratio was calculated as HC_10_/MIC or HC_10_/IC_50_. MIC values against reference strains were used for selectivity ratio calculation, unless denoted as (*). The most restrictive value (highest MIC or IC_50_ and lowest HC_10_) of an interval was chosen for selectivity ratio calculation. n.d.: non detectable activity.

Microorganism:	Peptide:	Oh-CATH	Bf-CATH	Bf-CATH30	Hc-CATH	Cdt-CATH	Ba-CATH	Pt-CATH1	∆Pb-CATH1	∆Pb-CATH2	∆Pb-CATH4	Pb-CATH1	Pb-CATH2	Pb-CATH3	Pb-CATH4	Pb-CATH5	Pb-CATH6	Sa-CATH
**Hemolysis (HC_10_)**																		
		~200	~31	50 - >400	>200	~104	~53	~13	~64	>64	~64	>100	>100	>100	<100	>100	>100	>200
**Gram-negative bacteria (MIC)**																		
*E. coli* ATCC 25922		0.25–8	8	2.3–8	2.3	0.25–0.78	0.25–0.78	2	2	3	1	9.4	37.5	n.d.	18.8	n.d.	n.d.	18.8
*E. coli* (CI)		2–20		0.6–16	2.3–9.4	16	16	16			4.7	9.4	75	n.d.	18.8	n.d.	n.d.	75
**Selectivity ratio**		25	4	6	>87	133	68	7	32	>21	64	>11	>3	-	<5	-	-	>11
**Gram-positive bacteria (MIC)**																		
*S. aureus* ATCC 25923		4–64	16	16 > 400	4.7–25.8	32	32	32	>128	>128	>128	37.5	n.d.	n.d.	18.8	n.d.	n.d.	75
*S. aureus* (CI)		8–64	32–64	16 > 400	4.7 > 200	32	32	32				4.7–37.5	75	n.d.	18.8	n.d.	n.d.	
**Selectivity ratio**		3	2	<0.1	>8	3	2	0.4	<0.5	0.5	<0.5	>3	-	-	<5	-	-	>3
**Fungi (MIC)**																		
*C. albicans* ATCC 2002				4.7														
*C. albicans* (CI)					2.3–4.7	10–40						9.4–18.8	18.8–37.5	n.d.	9.4–18.8	n.d.	n.d.	18.8–37.5
**Selectivity ratio**				11	43*	3*						>5*	>3*	-	<5*	-	-	>5*
**Tumor cells (IC_50_)**																		
PC-3 (prostate cancer)				70.2	n.d.													
U937 (leukemia)						<4												
MCF-7 (breast cancer)		n.d.		353							~64							
HepG2 (liver cancer)					n.d.													
**Selectivity ratio**		-		0.7	-	>26					1							
**Ref.**		[76,84]	[85]	[93,94,96,97]	[105,106]	[84,107,108,122,123]	[84]	[84]	[113]	[113]	[113]	[112]	[112]	[112]	[112]	[112]	[112]	[114]
**Color code:**		**Hemolysis:**			**Low (>50)**	**Medium (50–10)**				**High (>10)**								
		**Biological activity (MIC/IC_50_):**			**Low (>50)**	**Medium (50–10)**				**High (<10)**								
		**Non-selective (ratio < 1)**				**Selective (ratio > 1)**												

## Supplementary Materials

The following are available online at https://www.mdpi.com/2072-6651/12/4/255/s1: Table S1. SV-CATH precursors. SV-CATH protein precursors were collected, together with their sequences and domain information. Domains were highlighted in the protein sequence as: green, signal peptide; cathelicidin domain, blue; Glu-rich domain, yellow; mature peptide, red. Domain information (L, length; P, position) was extracted from NCBI (https://www.ncbi.nlm.nih.gov/protein). If no information was available (green background), domains were annotated based on bibliographic information and/or sequence similarities, Table S2. SV-defensins. Summary of SV-defensins identified to date, their sequences and accession numbers. A unified name was given to each SV-defensin according to the initial of the snake, Figure S1. Percentage identity matrix of SV-CATH precursors. Estimation of the homology/divergence between SV-CATH precursor proteins (sequences and NCBI accession numbers available in Table S1) illustrated by percentage identity matrix.

## Abbreviations

ACE	angiotensin-converting enzyme
ACP	anticancer peptide
AMP	antimicrobial peptide
CATH	cathelicidin
Ctn	crotalicidin
EC_50_	50% effective concentration
HC_10_	10% hemolytic concentration;
HPLC	high-performance liquid chromatography
IC_50_	50 % inhibitory concentration
L-AAO	L-amino acid oxidases
MRSA	methicillin-resistant *S. aureus*
MS	mass spectrometry
MSA	multiple sequence analysis
Naw	nawaprin
NMR	nuclear magnetic resonance
Omw	omwaprin
PLA_2_	phospholipases A2
SV	snake venom
