# Supplementary material for: Hitchhiking with Nature: Snake Venom Peptides to Fight Cancer and Superbugs
Source: Toxins (Basel). 2020 Apr 15;12(4):255. doi: 10.3390/toxins12040255 (PMC7232197; doi:10.3390/toxins12040255)
Supplement: Supplementary file 1 [file toxins-12-00255-s001.pdf]

Supplementary Materials: Hitchhiking with Nature: Snake Venom Peptides to Fight Cancer and Superbugs

Clara Pérez-Peinado, Sira Defaus and David Andreu

Table S1. SV-CATH precursors. SV-CATH protein precursors were collected, together with their sequences and domain information. Domains were highlighted in the protein sequence as: green, signal peptide; cathelicidin domain, blue; Glu-rich domain, yellow; mature peptide, red. Domain information (L, length; P, position) was extracted from NCBI (*https://www.ncbi.nlm.nih.gov/protein*). If no information was available (green background), domains were annotated based on bibliographic information and/or sequence similarities.

Source organism	Name	NCBI	Protein precursor sequence	Length	Signal peptide		Propeptide		Cathelicidin domain		Glu-rich domain		Mature peptide	
					L	P	L	P	L	P	L	P	L	P
Ophiophagus hannah	**Oh-CATH**	**B6S2X2.1**	**MEGFFWKTLLVVGALAIGGTSSLPHKPLTYEEAVDLAVSIYNSKSGEDSLYRLLEAVPPPEWDPLSESNQELNFTIKETVCLVAEERSLEECDFQEDGAIMGCTGYYFFGESPPVLVLTCKPVGEEEEQKQEEGNEEEKEVEKEEKEEDEKDQPRRVKRFKKFFKKLKNSVKKRAKKFFKKPRVIGVSIPF**	191	22	1–22	139	23–161	66	28–93	26	125–150	30	162–191
Bungarus fasciatus	Bf-CATH	B6D434.1	MEGFFWKTLLVVGALAIAGTSSLPHKPLIYEEAVDLAVSIYNSKSGEDSLYRLLEAVSPPKWDPLSESNQELNFTMKETVCLVAEERSLEECDFQEDGVVMGCTGYYFFGESPPVVVLTCKPVGEEGEQKQEEGNEEEKEVEEEEQEEDEKDQPRRVKRFKKFFRKLKKSVKKRAKEFFKKPRVIGVSIPF	191	22	1–22	139	23–161	66	28–93	26	125–150	30	162–191
Naja atra	Na-CATH	B6S2X0.1	MEGFFWKTLLVVGALTISGTSSFPHKPLTYEEAVDLAVSVYNSKSGEDSLYRLLEAVPALKWDALSESNQELNFSVKETVCQMAEERSLEECDFQEAGAVMGCTGYYFFGESPPVLVLTCKSVGNEEEQKQEEGNEEEKEVEKEEKEEDQKDQPKRVKRFKKFFKKLKNSVKKRAKKFFKKPKVIGVTFPF	191	22	1–22	139	23–161	66	28–93	24	125–148	30	162–191
Hydrophis cyanocinctus	Hc-CATH	AKJ54480.1	MQGFFWKTLLVVAALTIGGTSSLPHKPLTYEEAVDLAVSIYNSKSREEFLYRVLDAVPPPKWDPLSESNQELNFTIKETVCPVAEERSLEECGFQEDGAVMGCTGYFFFGESPPVLVLTCEPLVEEEQQKQEEGNEEEKEEKEEDEKDQPRRVKRFRKFFKRLLKSVRRAVKKFRKKPRLIGLSTLL	187	22	1–22	135	23–157	65	28–92	22	126–147	30	158–187

Crotalus durissus terrificus	Cdt-CATH	U5KJM4.1	MQGFFWKTWLVLAVCGTPASLAHRPLSYGEALELAVSVYNGKAGEASLYRLLEAVPQPEWDPSSEGSQQLNFTLKETACQVEEERSLEECGFQEDGVVLECTGYYFFGETPPVVVLSCVPVGGVEEEEEEEEEEQKAEAENDEEVEKEKGDEEKDQPKRVKRFKKFFKKVKKSVKKRLKKIFKKPMVIGVTIPF	194	22	1–22	142	23–164	65	26-90	29	125–153	34	161–194
Bothrops atrox	Ba-CATH	U5KJC9.1	MQGFFWKTWLVVALCGTSSSLAHRPLSYGEALELALSIYNSKAGEESLFRLLEAVPQPEWDPLSEGSQQLNFTIKETVCQVEEERPLEECGFQEDGVVLECTGYYFFGETPPVVVLTCVPVGGVEEEEEDEEEQKAEVEKDEEKEDEEKDRPKRVKRFKKFFKKLKNSVKKRVKKFFRKPRVIGVTFPF	189	22	1–22	133	23–155	65	26–90	24	125–148	34	156–189
Pseudonaja textilis	Pt-CATH1	U5KJJ1.1	MEGFFWKTWLVVAAFAIGGTSSLPHKPLTYEEAVDLAVSTYNGKSGEESLYRLLEAVPPPKWDPLSESNQELNLTIKETVCLVAEERSLEECDFQDDGAVMGCTGYFFFGESPPVLVLTCEPLGEDEEQNQEEEEEEEKEEDEKDQPRRVKRFKKFFMKLKKSVKKRVMKFFKKPMVIGVTFPF	184	22	1–22	128	23–150	66	28–93	12	132–143	34	151–184
	Pt-CATH2	U5KJM6.1	MDGFFWKTWLVVAALAIGGTSSLPHKPLTYEEAVDLAVSTYNGKSGEESLYRLLEAVPPPKWDPLSESNQELNLTIKETVCLVAEERSLEECDFQDDGAVMGCTGYFFFGESPPVLVLTCEPLGEDEEQNQEEEEEEEKEEDEKDQPRRVKRFKKFFRKLKKSVKKRVKKFFKKPRVIGVTIPF	184	22	1–22	128	23–150	66	28–93	12	132–143	34	151–184
Lachesis muta rhombeata	Lmr-CATH	U5KJZ2.1	MQGFFWKTWLVLAVCGTPASLAHRPLSYGEALELAVSVYNGKAGEASLYRLLEAVPQPEWDPSSEGSQQLNFTLKETACQVEEERSLEECGFQEDGVVLECTGYYFFGETPPVVVLSCVPVGGVEEEEEEEEEEQKAEAENDEEVEKEKEDEEKDQPKRVKRFKKFFKKVKKSVKKRLKKIFKKPMVIGVTFPF	194	22	1–22	138	23–160	65	26–90	29	125–153	34	161–194
Bothrops lutzi	Bl-CATH	U5KJT7.1	MQGFFWKTLLVVALCGTSSSLAHRPLSYGEALELALSVYNSKAGEESLFRLLEAVPQPEWDPLSEGSQQLNFTIKETVCQVEEERPLEECGFQEDGVVLECTGYYFFGETPPVVVLTCVPVGGVEEEEEDEEEQKAEVEKDEEKEDEEKDRPKRVKRFKKFFKKLKNNVKKRVKKFFRKPRVIGVTIPF	189	22	1–22	133	23–155	65	26–90	24	125–148	34	156–189
Python bivittatus	Pb1-CATH	AVI24168.1	MMEGCFWRILLVAGALSASGAAALPHRPLTYEEAVAFGVELYNKKAGEDSRYRLLEAVPQPDWDPTSESIQELNFTIKETVCLVQEERAEDECDFKDDGLVKECSGYYFFDETPPVAVLTCETVGGNEEETEEEKEEEKQPKRVKRFKKFFRKIKKGFRKIFKKTKIFIGGTIPI	175	23	1–23	121	24–144	80	27–106	11	129–139	31	145–175
	Pb2-CATH	AVR43560.1	MEIHPGRILLVLSLVVRGSVWAVEGEILSYDAALSLAVNLYNQESGWDVVFQLLEAKPQPEWDPSSKARQKLDFTLKETTCPTSQNLNLEVCDFKEQGVVVECSGSSLAQPGAPIIQFSCETATQGNHRVKRNGFRKFMRRLKKFFAGGGSSIAHIKLH	159	22	1–22	108	23–130	93	28–120	-	-	29	131–159
	Pb3-CATH	AVI24169.1	MMEGCFWRILLVAGALSASGAAPPPHKPLIYEKAVALGMELYNEKAGEDSQYRLLEAVPQPDWDPTSESTQELNFTIKETVCLVQEERAKDE	175	21	1–21	123	22–144	78	29–106	11	129–139	31	145–175

			CDFKDDGLVKECSGYYFFDETPPVAVLTCETVGGNEEETEEEKEEEKQPKRVKRFQNFFRELEKKFREFFRVYRITIGATIRF											
	Pb4-CATH	AVI24170.1	MTGVWALLLLLGVAAAAPPAQVVTYDQAIASAVNLYNQQKTTPFAFRLLEAEPQPNWDPRGKTTQGLKFTIKETVCPSAQSQNLTQCNFKEDGVDQDCSGTYSTQQEPPNLTVQCENIDQELNRITRSRWRRFIRGAGRFARRYGWRIALGLVG	154	16	1–16	109	17–125	98	21–118	-	-	29	126–154
	Pb5-CATH	AVR43562.1	MGLILLGAAWVALGILGSAASPTAEAPWLVLPRDAARLAVEDYNHRSPTPPSVFRLFKLRSTHKTRLEWGIHFSLHFTIKETHCQKTAGYRIGDCRYKPNGLIQDCSAEVSFLNLMWDSPVTSMKCGQAKWKKTKPHASPPQAMGFPPQVNVEHYIPASYSVAALTVTEEE	171	20	1–20	118	21–138	87	33–119	-	-	33	139–171
	Pb6-CATH	AVR43561.1	MHSFWVLLLFISPATTNFLSLSLTYPEALEEAVRLYNEEEGVKFLYRLVRAEPRPDWDPEAEGVQSLKFSMKETVCSAIEGLDFSKCDFKDDGEVKVCSASYKYQKKPQMNHVDVLCYCRQFCLFLFRQKAHTRLPVFRKSPHRFEAQAGQRSEGETGIPRPAMFRRPREGSKRAGGGRAGGPARPALRCHLEARRGRADVSGEARGLRRARAAPQRRLRAMARLKKFAEAGGADPDSGGLRARFPER	248	16	1–16	195	17–211	76	23–98	-	-	37	212–248
Sinonatrix annularis	Sa-CATH	QBZ68899.1	MEGCFWKILLVVGALTIGGTSTLAHKPLTYDEAVDLAVSIYNSKSGEDSLYRLLEAVPPSEWDPLSESNQDLNFTIKETVCQVAEERSLEECDFQEDGVVMECTGYYFFGETPPVLVLTCEAVGEEEEAEQQQEEGNGEEAEKEEKDEDKKDQPRRVKRFKKKFFKKLKKSVKKHVKKFFKKPKVIGVSIPF	191	24	1–24	133	25–157	78	28–105	17	126-142	34	158–191
Crotalus durissus cascavella	Cdc-CATH1	AGS36136.1	MEGSSGRPGWCWPSAGKEKEDEEKDQPKRVKRFKKKFFKKVKKSVKKRLKKIFKKPIFKKVKKSVKKRLKKIFKKPMVIGVTIPF	84	-	-	-	-	-	-	-	-	54	31–84
	Cdc-CATH2	AGS36137.1	MQGFFWKTLLVLAVCGTPASLAHRPLSYGEALELAVSVYNGKAGEASLYRLLEAVPQPEWDPSSEGSQQLNFTLKETACQVEEERSLEECGFQEDGDQPKRVKRFKKKFFKKVKKSVKKRLKKIFKKPMVIGVSIPF	136	22	1–22	80	23–102	65	26–90	-	-	34	103–136
	Cdc-CATH3	AGS36139.1	MQGFFWKTWLVLAVCGTPASLAHRPLSYGEALELAVSVYNGKAGEASLYRLLEAVPQPEWDPSSEGSQQLNFTLKETACQVEEERSLEECGFQEDGVVLECTGYYFFGETPPVVVLSCVPVGGVEEEEEEEEEEQKAEAENDEEVEKEKGDEEKDQPKRVKRFKKKFFKKVKKSVKKRLKKIFKKPMVIGVTIPF	194	22	1–22	138	23–160	65	26–90	29	125–153	34	161–194
Thamnophis sirtalis	Ts-CATH1	XP_013912467.1	MEGCFWKALLVVGALAIGGTSTLAHKPLTYDEAVNLAVSTYNNKSGEGTLYRLLEAVPPPEWDPLSEGNQELNFTIKETVCKVGEELSLEECSFQEDGAVMECTAYFFFGEKPPLLVLTCEAVSEEEQQEEEEEGNEEEKEAGKEEDEKDQPRRVKRFKKKFFKKIKKSVKKRVKKLFKKPRVIPISIPF	189	24	1–24	131	25–155	65	28–92	24	125–148	34	156–189

	Ts-CATH2	XP_013917356.1	MDGFFWKIWLAVGALTIGGTSSLPRKPLTYDKAVELGVAIYNSKAG<br>EDSLYRLLEAVPQPEWDPYSESYQELNFTIKQTVCPVEEEFSTDECDF<br>KENGLVRQCTGYYFLEERPPVAVLTCDTVGGTAEEEEKEKKEEEVVE<br>KEEEEEEEKERRDFC	156	22	1–22	-	-	66	28–93	24	128–151	-	-
	Ts-CATH3	XP_013928483.1	MEGFLWKTLLLVGALSASGRSAPSPKLLTYDEAVVQAVVNYNGKA<br>KEGSLYQLLEAAPQPDWDPNFEGTQELKFTIKETVCRAEEEGSLDKC<br>DFKEDGVVRDCTATYFLGEKTPVAFLDCKAVGETEEEEEEVEEKEEG<br>TEEEESVERSRKRIKKRRRIRVQITVKITFKI	171	21	1–21	131	22–153	66	28–93	22	126–147	18	154–171
	Ts-CATH4	XP_013912465.1	MEGSFWKTWLVVGALLVFGCSSLPHKPLTYEKAVDLAVAIYNSKAG<br>EDCVYRLLEALAEPQWDPISDSHEELNFTIKETMCLLEDVVFFDECD<br>FKEDGVIRQCTGYYYFDERPPVVVLTCVVVAGMEEEKGEEVGKKEE<br>EKQEEEEEKEEENQARNKEEEEKKEEEEEKEEGKETEKQEEKEEEEKL<br>KKGLKKLFKRKKVVAGYVTA	207	22	1–22	165	23–187	54	28–81	59	127–185	20	188–207
Protobothrops mucrosquamatus	Pm-CATH	XP_015682911.1	MEGFFWKTLMLVGVLSVSGRPHLPHEPLTYDDALCLGVEIFNKKAG<br>EGSLYRLLDGVPQPEWDPVSEGNQALNFTVKETVCPAEEDFSTDRC<br>DFKEDGMVRQCTGYYFLEERPPVAVLTCHTVGGTVKEEEKEEEEEK<br>KKKKDQPKRVKRFAGFFQFVVGVSFRF	165	19	1–19	129	20–148	66	28–93	9	129–137	17	149–165

Table S2. SV-defensins. Summary of SV-defensins identified to date, their sequences and accession numbers. A unified name was given to each SV-defensin according to the initial of the snake.

Snake	Unified name	Other name	NCBI	Peptide sequence	Length	Ref
Phalotris mertens	**Pm1-defensin**	**-**	**JAS03136.2**	**MKILYLLFALLFLAFLSEPGNAQPRCHSQGGRCYFLLCPWNTIDHGQLDCPGTRICCEPKCPGK**	**64**	[1]
	Pm2-defensin	-	JAS03135.2	MKILYLLFALLFLAFLSEPGNAQRICLGGRGFCHSTPCPRSTIDYGKKDCWGSLRCCEPKRPGK	64	[1]
Crotalus oreganus helleri	Coh1-defensin	Beta_Defensin-CohLL-1	JAA98041.1	MKILYLLFAFLFLAFLSEPGNAYKRCHKKGGHCFPKTVICLPPSSDFGKMDCRWKWKCCKKGSVNNAISI	70	NCBI
	Coh2-defensin	Beta_Defensin-CohPH-6	JAA98013.1	MKILYLLFAFLFLAFLSEPGNAYKRCHKKGGHCFPKEKICIPPSSDFGKMDCRWKWKCCKREVENNAISI	70	NCBI
	Coh3-defensin	Beta_Defensin-CohID-3	JAA97983.1	MKILYLLFAFLFLAFLSEPGNAYKRCLKKGGHCFPKTVICLPPSSDFGKMDCRWKWKCCKKGSVNNAISI	70	NCBI
Crotalus durissus terrificus	Cdt-defensin	Crotasin	AAT47437.1	MKILYLLSAFLFLAFLSESGNAQPQCRWLDGFCHSSPCPSGTTSIGQQDCLWYESCCIPRYEK	63	[2]
Lachesis muta	Lm1-defensin	beta-defensin-like protein	AGF25395.1	MKILYLLFPFLFLAFLSEPGNAQGQCHQQRGRCFLHQCPLSHYFLGRLDCGPGRRCCRRRK	61	[3]
	Lm2-defensin	beta-defensin-like protein	AGF25394.1	MKILYLLFPFLFLAFLSEPGNAQEWCRGLGGFCSFYQCRPGHDLGPQDCWPERRCCRWGK	60	[3]
Bothrops pauloensis	Bp-defensin	beta-defensin-like protein	AGF25393.1	MKILYLLFTFPFLAFLSEPGNAQPECLRQGGMCRPRPCPYVSLGHLDCQMGQMCCIRKPRK	61	[3]
Bothrops neuwiedi	Bn-defensin	beta-defensin-like protein	AGF25392.1	MKILYLLFTFLFLAFLSEPGNAQPECCQEGGICHSKQCPLGYSSLGRLDCQLGQRCCIRIFGK	63	[3]
Bothrops matogrossensis	Bm1-defensin	beta-defensin-like protein	AGF25391.1	MKILYLLFTFLFLAFLSEPGNAQRRCRQRRGICRPRPCPPENFSLGRLDCQMGQMCCRRRFGK	63	[3]
	Bm2-defensin	beta-defensin-like protein	AGF25390.1	MKILYLLFTFLFLAFLSEPGNAQRRCRQRRGICRPRPCPPENFSLGRLDCQMGRMCCRRRFGK	63	[3]

Bothrops leucurus	Bl-defensin	beta-defensin-like protein	AGF25389.1	MKILYLLFTFLFLAFLSEPGNAQRRCRQKGGMCLPGPCPPGYVSLGQQDCRRGQMCCIRFGK	62	[3]
Bothrops jararacussu	Bja-defensin	beta-defensin-like protein	AGF25388.1	MKILYLLFTFLFLAFLSEPGNAQRRCHQKGGMCLPGPCPPGYDSLGQQDCRRGQKCCIKRFGK	63	[3]
Bothrops jararaca	Bj1-defensin	beta-defensin-like protein Defb_Bj-03	AVN97891.1	MKILYLLFTFLFLAFLSEPGNAQPECCQQGGICHSKQCPLGYSSLGRLDCQLGQRCCIRIFGK	63	[4]
	Bj2-defensin	beta-defensin-like protein	AGF25387.1	MKILYLLFTFLFLAFLSEPGNAQVRCRRLGGICILSRCPLRYDSLGQQDCLKGQKCCRRRFGK	63	[3]
	Bj3-defensin	beta-defensin-like protein	AGF25386.1	MKILYLLFTFLFLAFLSEPGNAQEECLQQGGFCRLIRCPFGYDSPEQQDCRKGQRCCIRKPRK	63	[3]
Bothrops erythromelas	Be-defensin	beta-defensin-like protein	AGF25385.1	MKILYLLFTFLFLAFLSEPGNAQEGCLQQGGFCRLIRCPFGYDSLEQQDCRKGQRCCIRKPRK	63	[3]
Bothrops diporus	Bd-defensin	beta-defensin-like protein	AGF25384.1	MKILYLLFTFLFLAFLSEPGNAQPECLRQGGMCRPRLCPYVSLGQLDCQNGHVCCRKKPRK	61	[3]
Bothrops atrox	Ba-defensin	beta-defensin-like protein	AGF25383.1	MKILYLLFTFLFLAFLSEPGNAQRECYWQRGFCRSKGCPFGYDSLGRLDCPLGYVCCRI	59	[3]
Thamnophis siralis	Ts-defensin	-	XR_001313470.1	MKIFYLLFAFLFLAFLSEPGSAQSLCHRKRGRCLEICPKGTKDIHRWDCRHGLTCCVPARGK	62	NCBI
Protobothrops mucrosqumatus	Pmu-defensin	crotasin-like	XM_015824769.1	MKILYLLFTFLFLAFLSEPGNAQPPECRNIGGQCYQSRCPDGKNNNGWKDCEWGQLCCNRKW KIMPSPSTTMDTIKIWPMT	81	NCBI
Crotalus adamanteus	Ca-defensin	myotoxin	HQ414100.1	MKILYLLFAFLFLAFLSEPGNAYKRCHKKGGHCFPKTVICLPPSSDFGKMDCRWRWKCCKKGV	63	[5]
Ovophis okinavensis	Oo-defensin	crotasin-like_protein	AB852005.1	MKILYLLFAFLFLAFLSEPGNAQGQCYRPGGLCLSDPCPSGHLDLGQVDCQPGQKCCRRGSGK	63	[6]

	Pb-CATH5	Cdc-CATH1	Ts-CATH3	Pb-CATH1	Pb-CATH3	Ts-CATH4	Pm-CATH	Ts-CATH2	Ba-CATH	Bl-CATH	Cdc-CATH2	Cdc-CATH3	Cdt-CATH	Lmr-CATH	Ts-CATH1	Hc-CATH	Sa-CATH	Pt-CATH1	Pt-CATH2	Na-CATH	Bf-CATH	Oh-CATH	Pb-CATH6	Pb-CATH4	Pb-CATH2
**Pb-CATH5**	100																								
**Cdc-CATH1**	12.9	100																							
**Ts-CATH3**	20.27	31.03	100																						
**Pb-CATH1**	21.38	66.67	52.83	100																					
**Pb-CATH3**	21.38	47.62	53.46	84.57	100																				
**Ts-CATH4**	20	27.42	44.91	52.63	51.46	100																			
**Pm-CATH**	19.87	56.67	55.62	58.12	57.5	47.24	100																		
**Ts-CATH2**	20.42	35.29	57.24	64.34	64.34	61.29	67.57	100																	
**Ba-CATH**	19.38	75	48.8	59.65	56.73	48.66	58.28	63.4	100																
**Bl-CATH**	19.38	75	50	60.82	57.89	47.59	57.67	62.09	97.88	100															
**Cdc-CATH2**	23.33	97.5	50.85	61.36	53.03	45.52	61.34	58.82	83.09	84.56	100														
**Cdc-CATH3**	20	83.93	49.1	62.57	56.14	49.48	58.9	57.79	85.19	85.19	98.53	100													
**Cdt-CATH**	20	83.93	49.1	62.57	56.14	49.48	58.9	57.79	85.19	85.19	98.53	100	100												
**Lmr-CATH**	20	83.93	49.1	62.57	56.14	50	59.51	58.44	86.24	85.19	97.79	98.97	98.97	100											
**Ts-CATH1**	21.6	69.23	52.41	60.12	54.34	49.2	53.94	58.28	66.12	66.67	72.06	65.78	65.78	65.78	100										
**Hc-CATH**	20.37	54	46.39	55.49	53.18	47.03	52.12	61.59	64.48	63.93	65.44	60	60	60.54	71.51	100									
**Sa-CATH**	21.6	69.23	52.38	63.58	59.54	50.26	58.18	63.4	71.35	71.35	73.53	67.72	67.72	67.2	78.84	74.33	100								
**Pt-CATH1**	20.99	77.55	50.61	62.43	58.38	50.55	53.94	61.74	71.98	70.33	72.06	70.33	70.33	71.43	76.63	79.89	79.35	100							
**Pt-CATH2**	20.99	79.59	51.22	63.58	59.54	51.1	53.33	63.09	72.53	71.98	72.79	70.88	70.88	70.88	78.26	81.52	80.43	96.74	100						
**Na-CATH**	22.22	73.08	51.19	60.69	57.23	51.85	55.76	62.09	67.57	67.57	69.85	64.55	64.55	65.61	69.31	76.47	78.01	80.43	80.43	100					
**Bf-CATH**	23.46	69.23	52.38	63.01	61.27	54.5	56.36	64.71	68.65	68.65	72.06	66.67	66.67	66.67	73.54	79.14	79.06	8.,7	85.87	84.82	100				
**Oh-CATH**	23.46	73.08	53.57	64.16	60.12	56.08	57.58	67.32	71.89	71.89	75	67.72	67.72	67.72	77.78	83.42	83.25	86.41	88.04	89.01	92.67	100			
**Pb-CATH6**	19.87	14.29	34.84	30.95	30.95	22.62	33.12	30.66	29.76	29.76	34.62	29.76	29.76	29.76	29.41	27.65	28.82	29.41	28.82	28.82	28.82	28.82	100		
**Pb-CATH4**	22.3	6.9	39.29	35.33	34.67	30.26	29.66	34.33	30.26	30.26	31.62	29.61	29.61	29.61	29.87	31.82	32.47	31.17	32.47	27.92	30.52	31.82	35.53	100	
**Pb-CATH2**	21.57	24.14	30.99	33.33	31.37	27.27	32.65	33.82	35.71	35.06	42.02	37.66	37.66	37.66	33.33	29.49	34.62	30.77	30.77	30.13	31.41	32.05	28.76	39.61	100

**% Identity:**	<50	50–70	70–90	>90

Figure S1. Percentage identity matrix of SV-CATH precursors. Estimation of the homology/divergence between SV-CATH precursor proteins (sequences and NCBI accession numbers available in Table S1) illustrated by percentage identity matrix.

References:

1. Campos, P.F.; Andrade-Silva, D.; Zelanis, A.; Paes Leme, A.F.; Rocha, M.M.; Menezes, M.C.; Serrano, S.M.; Junqueira-de-Azevedo Ide, L. Trends in the Evolution of Snake Toxins Underscored by an Integrative Omics Approach to Profile the Venom of the Colubrid Phalotris mertensi. *Genome Biol. Evol.* **2016**, *8*, 2266–2287.
2. Radis-Baptista, G.; Kubo, T.; Oguiura, N.; Prieto da Silva, A.R.; Hayashi, M.A.; Oliveira, E.B.; Yamane, T. Identification of crotasin, a crotamine-related gene of Crotalus durissus terrificus. *Toxicon* **2004**, *43*, 751–759.
3. Correa, P. G.; Oguiura, N., Phylogenetic analysis of beta-defensin-like genes of Bothrops, Crotalus and Lachesis snakes. *Toxicon* **2013,** *69*, 65-74.
4. de Oliveira, Y. S.; Correa, P. G.; Oguiura, N., Beta-defensin genes of the Colubridae snakes Phalotris mertensi, Thamnodynastes hypoconia, and T. strigatus. *Toxicon* **2018,** *146*, 124-128.
5. Rokyta, D.R.; Wray, K.P.; Lemmon, A.R.; Lemmon, E.M.; Caudle, S.B. A high-throughput venom-gland transcriptome for the Eastern Diamondback Rattlesnake (Crotalus adamanteus) and evidence for pervasive positive selection across toxin classes. *Toxicon* **2011**, *57*, 657–671.
6. Aird, S.D.; Watanabe, Y.; Villar-Briones, A.; Roy, M.C.; Terada, K.; Mikheyev, A.S. Quantitative high-throughput profiling of snake venom gland transcriptomes and proteomes (Ovophis okinavensis and Protobothrops flavoviridis). *BMC Genom.* **2013**, *14*, 790.
